# Neurotensin inhibits AMPK activity and concurrently enhances FABP1 expression in small intestinal epithelial cells associated with obesity and aging

**DOI:** 10.1038/s12276-025-01461-w

**Published:** 2025-06-02

**Authors:** Jing Li, Jun Song, Baoxiang Yan, Haoming Wu, Moumita Banerjee, Leif Magnuson, Yajuan Liu, Shulin Zhang, Jinpeng Liu, Chi Wang, Tianyan Gao, Jianhang Jia, Heidi L. Weiss, B. Mark Evers

**Affiliations:** University of Kentucky, Lexington, KY USA

**Keywords:** Cancer metabolism, Colon cancer

## Abstract

We previously demonstrated that neurotensin, a 13-amino-acid gut hormone peptide, enhances small intestinal epithelial cell fatty acid uptake through inhibition of AMPK. Here, utilizing *Drosophila* and mouse models in vivo, as well as mouse and human small intestinal epithelial organoids or monolayers ex vivo, we determine the targets of neurotensin and AMPK associated with obesity and aging. High-fat diet and aging decreased AMPK and insulin signaling, which was prevented by neurotensin deficiency. High-fat diet feeding increased FABP1 protein expression in wild-type mice; this effect was attenuated in neurotensin-deficient mice. AICAR and metformin increased AMPK phosphorylation in young but not in aged small intestinal epithelial cells. By contrast, AICAR and metformin inhibited FABP1 mRNA and protein expression. Moreover, cytosolic colocalization of AMPKα1 and FABP1 was noted in IEC-6 cells. AMPK phosphorylation and FABP1 expression was decreased in aged wild-type small intestinal epithelial cells; however, this effect was reversed in neurotensin-deficient cells. Results from human duodenal organoids confirm the effects of neurotensin, palmitic acid and metformin on AMPK phosphorylation and FABP1. Finally, overexpressing neurotensin in enteroendocrine cells reduced the lifespan of *Drosophila*; neurotensin deficiency extended the lifespan of mice fed a high-fat diet. Our findings indicate that neurotensin inhibits AMPK and increases FABP1 in small intestinal epithelial cells under conditions of obesity. Neurotensin deficiency preserves AMPK and FABP1 levels, thus attenuating some of the negative effects of obesity and aging.

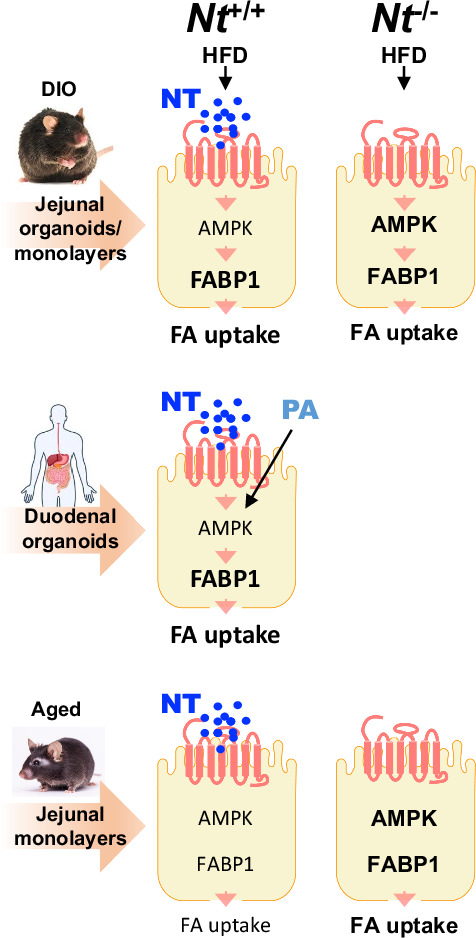

## Neurotensin impacts fatty acid uptake in aging cells

Neurotensin (NTS) is a hormone primarily localized to the gut and brain that affects various bodily functions. Researchers have found that high levels of a form of NTS, called pro-NTS, are linked to diabetes and heart problems. Researchers used mice and fruit flies to study NTS’s role in fat absorption during obesity and aging. They found that NTS can reduce the activity of a protein called AMPK, which helps to control energy use in cells. In mice lacking NTS, AMPK activity was higher, leading to less fat absorption and better insulin function. This suggests that NTS might contribute to obesity by lowering AMPK activity. The study also showed that reducing NTS levels could improve health and extend lifespan in mice. These findings suggest that targeting NTS could help to manage obesity and related health issues.

This summary was initially drafted using artificial intelligence, then revised and fact-checked by the author.

## Introduction

Neurotensin (NTS), a 13-amino acid peptide discovered by Carraway and Leeman^1^, is mainly synthesized by N cells of the small intestine and released into the bloodstream acting as a circulating hormone or in the brain as a peptidergic neurotransmitter or neuromodulator^2^. The physiological effects of NTS are through its receptors (NTSRs), including NTSR1, NTSR2 and NTSR3 (also called sortilin or SORT1). In a landmark study published in 2012, Melander et al.^3^ showed that increased fasting levels of pro-NTS (the final stable 117-amino-acid polypeptide after the NTS precursor is cleaved by convertases^4^) were associated with the development of diabetes and an increased risk of cardiovascular disease and mortality, thus providing the first clinical evidence that excessive NTS secretion results in metabolic disorders and increased morbidity and mortality. More recently, pro-NTS has been shown to be a novel diagnostic biomarker for detection of fatty liver disease^5^ and type 2 diabetes mellitus^6^. Previously, we reported that NTS deficiency protected against body weight (BW) gain, insulin resistance and hepatic steatosis associated with the consumption of a high-fat diet (HFD)^7^. We further showed that NTS stimulates fatty acid (FA) absorption in the small intestine by attenuating the activation of AMP-activated protein kinase (AMPK) and involving both NTSR1 and NTSR3. In complementary studies, we demonstrated increased lipid accumulation in transgenic *Drosophila* strains expressing NTS specifically in gut enteroendocrine (EE) cells. Remarkably, in adults, we showed that increased levels of pro-NTS strongly predict new-onset obesity in a graded manner, which is independent of body mass index and insulin resistance.

AMPK exists as a complex consisting of an α catalytic subunit (α1 or α2) and regulatory subunits β (β1 or β2) and γ (γ1 or γ2 or γ3)^8,9^. Reznick et al.^10^ found that stimulation of AMPKα2 by 5′-aminoimidazole-4-carboxamide-1-β-d-ribofuranoside (AICAR), an AMPK activator, was blunted in the skeletal muscle of aged versus young rats. AMPK activation in the muscle or fat can extend lifespan in *Drosophila*^11^. Furthermore, AMPK upregulation in *Drosophila* demonstrates both autonomous effects on intestinal epithelium and nonautonomous effects on distant tissues, such as the brain, linked to reduced insulin-like peptide levels and increased 4E-BP expression, which collectively slow systemic aging, thus demonstrating that localized activation of AMPK in key tissues can slow aging in a non-cell-autonomous manner^12^.

With obesity, AMPK activity is generally reduced in tissues such as skeletal muscle, liver and adipose tissue^13^. Basal AMPK activity is generally higher in young muscle versus aged muscle^14,15^. Young cells can efficiently activate AMPK in response to metabolic stress such as exercise, fasting or caloric restriction. However, basal AMPK activity is lower in aged skeletal muscle. The ability of cells to activate AMPK in response to stressors is diminished with age^16^. This can lead to a decline in mitochondrial function, decreased energy production and increased susceptibility to metabolic disorders. The importance of AMPK in the intestine has been recognized only recently. Both AICAR and metformin (Met), another AMPK activator, control glucose metabolism in the intestine and maintain whole-body glucose homeostasis^17^. Intraduodenal infusion of Met activated duodenal mucosal AMPK and lowered hepatic glucose production in a HFD-induced rat model with insulin resistance^18^. Mice with knockout (KO) of intestinal epithelium-specific AMPKα1 gained weight, and glucose tolerance was impaired compared with wild-type (WT) mice after 6 weeks of HFD feeding^19^. Intestinal epithelium-specific AMPKα1 deletion impaired intestinal long-chain FA absorption and protected mice from HFD-induced obesity^20^, suggesting the involvement of AMPK in intestinal FA absorption.

FA binding protein 1 (FABP1, also called liver FABP or L-FABP) and FABP2 (also called intestinal FABP or I-FABP) are expressed in enterocytes, but not in crypt cells^21^. Both proteins are highly expressed in the jejunum, the major site of absorption of dietary lipids^22^, compared with the duodenum, ileum and colon^21^. FABP1 and FABP2, both of which bind long-chain FAs^23^, have different functional effects in the intestine^24^. FABP1 regulates genes involved in FA oxidation and the formation of prechylomicron transport vesicles^25^. By contrast, apical cytoplasmic FABP2 binds FAs from the mucosa of fasting rats and transports the FAs into the interior of the cell, suggesting that FABP2 is more likely to be involved in FA uptake from the lumen of the intestine and with the distribution of FAs to metabolic compartments^26^. Utilizing FABP1- and FABP2-deficient mice, Lagakos et al.^24^ found that FABP1 directs FAs toward oxidative pathways, while FAPB2 targets dietary FAs toward triglyceride synthesis.

In the present study, FABP1 was upregulated by HFD feeding, which correlates with the HFD-induced inhibition of AMPK activity. Consistently, Met or AICAR activated AMPK and concurrently decreased FABP1 protein expression, further supporting the role of AMPK in controlling intestinal lipid absorption. NTS deficiency restores AMPK signaling as well as FABP1 impaired by HFD feeding and aging in IECs intestinal epithelial cells.

## Materials and methods

### Reagents

Phospho-AMPKα (Thr172) (2535), AMPKα (2532), phospho-ERK1/2 (Thr202/Tyr204) (4370), ERK1/2 (9102), phospho-Akt (Ser-473) (4058) and pan-Akt (4691) antibodies were from Cell Signaling Technology. AMPKα1 (sc19128), AMPKα2 (sc19129), FABP1 (sc-374537) and FABP2 (sc-374482) antibodies were from Santa Cruz Biotechnology. GFP (ab290) and RFP (ab62341) antibodies were from Abcam. AICAR, Met and PD 98059 were from Cayman. Fetal bovine serum, NTS_1–13_, human insulin, palmitic acid (PA), *N*-acetyl cysteine and β-actin antibody were from Sigma-Aldrich. SYBR green primers for real-time or quantitative PCR (qPCR) were from Integrated DNA Technologies. Noggin-conditioned medium (CM) was purchased from U-Protein Express BV. Dulbecco’s modified Eagle medium (DMEM), advanced DMEM/F12 medium, OptiMEM reduced serum medium, growth factor-reduced Matrigel, B-27 supplement, N-2 supplement, HEPES, GlutaMAX and Zeocin were from ThermoFisher. Mouse EGF was from PeproTech. ChromoTek GFP-Trap agarose was from Proteintech.

### Cell culture and stable cell lines

The normal rat small intestinal epithelial cell line IEC-6 and the human embryonic kidney cell line HEK-293, purchased from ATCC, were maintained in DMEM, supplemented with 10% fetal bovine serum. pEGFP-N1 was a gift from Antony K. Chen (Addgene plasmid #172281)^27^. mRFP1-N1 was a gift from Robert Campbell, Michael Davidson and Roger Tsien (Addgene plasmid #54635)^28^. Human NTSR1 and NTSR3 cDNAs were cloned into pEGFP-N1 and mRFP1-N1, respectively. HEK-293 cells were transfected with constructs by LipoFectamine 3000 (ThermoFisher). Stable cell lines (293/GFP), 293/GFP-NTSR1 and 293/RFP-NTSR3) were selected with G418 (800 μg/ml), sorted by flow cytometry and maintained in culture medium containing 500 μg/ml G418.

### Assessment of *Drosophila* lifespan

*Gr36C*-Gal4 and the NTS transgenic lines have been previously described^7^. The EE-cell-specific *Gr36C*-Gal4 was used to drive NTS expression. *Gr36C*-NTS flies were back crossed with w1118 for eight generations to synchronize the genetic backgrounds. Flies were housed in vials at 25 °C and provided with normal food, and their lifespans were monitored regularly by counting the number of surviving flies. Survival flies were counted at the days after adult eclosion. Male and female flies were separately cultured and counted.

### Assessment of mouse lifespan and EchoMRI

*Nts*^+/+^ and *Nts*^−/−^ mice were placed on normal chow (NC) or 60% HFD at weaning for as long as they survived. The body fat and lean mass of aged mice was assessed by EchoMRI as previously described^7^.

### Western blot analysis and immunoprecipitation

Protein preparation and western blotting were performed as described previously^29,30^. In brief, cells were lysed with lysis buffer (Cell Signaling Technology) and equal amounts of protein were loaded into 4–12% NuPAGE BisTris gels (ThermoFisher) and transferred via electrophoresis to polyvinylidene difluoride membranes; the membranes were incubated with primary antibodies overnight at 4 °C followed by secondary antibodies conjugated with horseradish peroxidase. Membranes were developed using Amersham ECL Western Blotting Detection Reagent from GE Healthcare Life Sciences or Immobilon Western Chemiluminescent HRP substrate from ThermoFisher. For immunoprecipitation (IP), cells were cultured in 100-mm dishes and GFP or RFP was pulled down with ChromoTek GFP-Trap agarose followed by western blot analysis.

### RNA isolation, qPCR and RNA-seq analysis

Total RNA was purified using RNeasy Mini Kit following the manufacturer’s instructions (Qiagen), and cDNA was synthesized using High-Capacity cDNA Reverse Transcription Kit (Applied Biosystems). qPCR was performed using a SYBR Green Universal Master Mix. Mouse β-actin and human β-actin were used as the internal controls. Expression levels were assessed by evaluating threshold cycle (Ct) values. The relative amount of mRNA expression was calculated by the comparative ΔΔCt method.

Female *Nt*^+/+^ and *Nt*^−/−^ mice were fed either a 60% HFD or 10% low-fat diet (LFD) at weaning for 28 weeks. Mice were fasted overnight, and jejunal mucosal RNA was purified as previously described^7^ and sent to the University of Kentucky Markey Cancer Center Oncogenomics Shared Resource for RNA-seq. RNA-seq libraries were prepared using KAPA RNA Hyper + RiboErase HMR (Roche). The manufacturer’s protocols were used to sequence ribosomal RNA-depleted libraries at 1 × 100 single-end reads on an Illumina HiSeq 2500 (or other sequencer) in rapid mode, to an average depth of 40 × 10^6^ paired-end reads per sample. The data were stored as FASTQ files.

### Immunofluorescence staining and confocal microscopy

Immunofluorescence (IF) staining was performed as described previously^29^. In brief, cells were cultured on glass coverslips in 24-well plates. Forty-eight hours after plating, cells were fixed with 4% paraformaldehyde and stained with primary antibodies followed by Alexa Fluor 488 secondary antibody. Nuclei were counterstained with Hoechst. Images were obtained using a Nikon A1 confocal on an Eclipse Ti2 microscope with a 40×, or 100× 1.35-numerical-aperture oil objective.

### Mice

All procedures were approved by the Institutional Animal Care and Use Committee of the University of Kentucky. *Nts*^−/−^ and *Ntsr1*^−/−^ mice and their WT littermates (*Nts*^+/+^ and *Ntsr1*^+/+^) were bred from *Nts*^*+/−*^ or *Ntsr1*^+/−^ mice and randomly grouped for all experiments. Mice were maintained with a 14 h light/10 h dark cycle and provided with food and water ad libitum. For diet-induced obesity, male and female *Nts*^+/+^ and *Nts*^−/−^ mice were placed on a 60% HFD or 10% LFD (cat. nos. D12492 and D12450B, respectively; Research Diets) at weaning as we previously reported^7^. Aged mice were maintained on NC for 12 or 20 months, and BW was measured. Young and aged mice were purchased from ´The Jackson Laboratory.

### Mouse jejunal crypt isolation and organoid culture

Small intestinal crypts were isolated from mice as previously described with modifications^31^. In brief, the crypts were isolated from jejunal fragments with 2 mM EDTA–phosphate-buffered saline (PBS) (pH 7.4), embedded in Matrigel and overlaid by ENR medium (advanced DMEM/F12 supplemented with B-27, N2, *N*-acetyl cysteine, HEPES, GlutaMAX (basal medium) containing EGF (50 ng/ml), noggin CM (1:100) and R-spondin-1 CM (1:20)). Mature organoids were passaged every 4–7 days. For PA treatment, mature organoids were passaged and cultured in ENR medium supplemented with PA (30 μM) for 7 days. For R-spondin CM, Cultrex HA-R-Spondin1-Fc 293T cells, purchased from Trevigen, were cultured following the manufacturer’s instructions.

### Mouse jejunal monolayer culture

Jejunal crypts were isolated as described above, and monolayer cultures were established as described previously^32^ with slight modifications. In brief, freshly isolated crypts were cultured in plates precoated with 2% Matrigel and ENR medium containing 10 μM blebbistatin (B-ENR medium). The medium was replaced with basal medium containing 10 μM blebbistatin, 0.5 μM LDN-193189, 2.5 μM CHIR-99021 and 10% R-spondin CM. Treatments were started concurrently with the medium changes.

### Human duodenal organoid culture

Approximately 2–3 cm of normal human duodenal tissue was obtained from the Biospecimen Procurement and Translational Pathology shared resource facility of the University of Kentucky Markey Cancer Center after surgical resection. All human tissues were deidentified and obtained following approval by the University of Kentucky Institutional Review Board. Crypts were isolated as described previously^33^ with slight modifications. In brief, the mucosal surface was gently scraped with a glass slide, washed with ice-cold PBS and then incubated in PBS with 2 mM EDTA on ice for 30 min on a shaker with three-dimensional rotation. The mucosa was scraped in 0.1% BSA–PBS to release the crypts using a glass slide. Crypts were suspended in Matrigel and cultured in 1:1 mixed basal medium with L-WRN CM with addition of 50 ng/ml EGF, 500 nM A-83-01, 10 μM SB202190, 10 mM nicotinamide, 10 nM [Leu]15-gastrin, 2.5 μM CHIR-99021 and 2.5 μM thiazovivin (WENRNicASGCT medium). Organoids were passaged every 7–10 days. L-WRN cells, purchased from ATCC, were used to make L-WRN CM as previously reported^34^.

### Statistical analysis

Descriptive statistics (mean and standard deviation) for each experimental condition were calculated and presented in bar graphs. Comparisons of in vivo studies measuring mRNA, densitometry levels and BW were performed between genotype, diet, animal sex and age groups using two-way analysis of variance (ANOVA) with two-way interaction between factors. Pairwise comparisons between groups were performed using contrast statements in the ANOVA model with adjustments for multiple testing using Holm’s *P*-value adjustment. Human samples measuring the FABP1/FABP2 ratio were summarized descriptively. For the *Drosophila* studies, statistical analysis was performed using the Student’s two-sample *t*-test on the lifespan data to determine if there are significant differences in lifespan among the NTS-expressing flies and the *w1118* WT flies. Normality and heterogeneity of variance assumptions were evaluated to determine the validity of the parametric tests and models utilized. In vivo studies on survival were analyzed using the Kaplan–Meier estimate for each genotype and diet groups with comparison of survival curves using the Wilcoxon–Gehan test.

## Results

### NTSR1, which forms a complex with NTSR3, mediates NTS-inhibited AMPK signaling

**Fig. 1:**
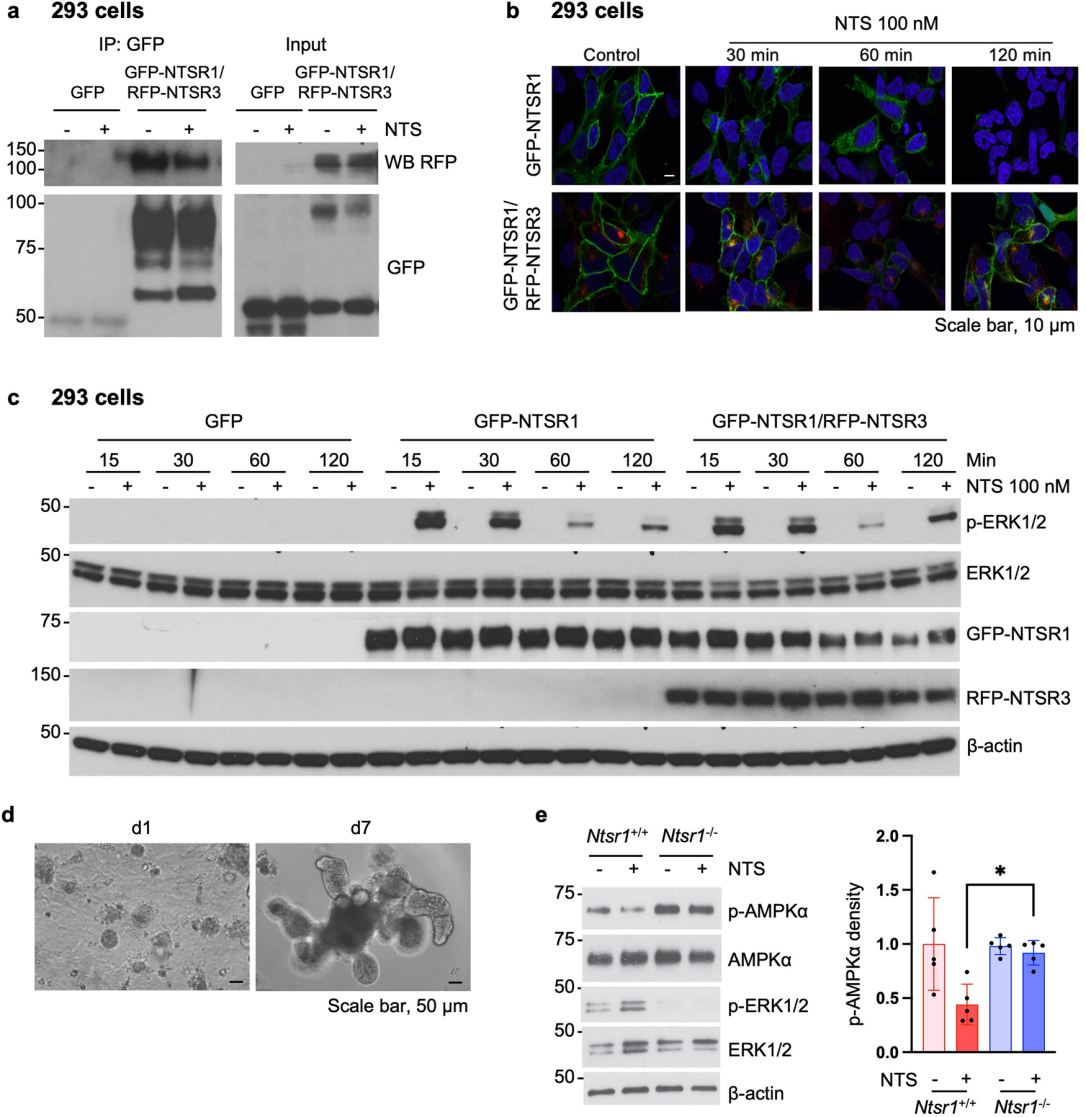
NTSR1, which forms a complex with NTSR3, mediates AMPK activity. **a–c** Stable 293 cell lines overexpressing GFP, GFP-tagged NTSR1 or combined with RFP-tagged NTSR3 were established. Cells were treated with or without NTS (100 nM) for 15 min followed by IP assay using anti-GFP antibody followed by western blot analysis using anti-GFP and RFP antibodies (**a**); cells were treated with NTS (100 nM) for different lengths of time followed by confocal microscopic analysis (**b**) or western blot analysis (**c**). **d** Mouse jejunal organoid model. Images were taken at day (d)1 and d7 by inverted microscope. **e** Western blot analysis of protein extracted from jejunal organoids of *Ntsr1*^+/+^ and *Ntsr1*^−/−^ mice; cells were treated with or without NTS (10 nM) for 10 min. Densitometric analysis of p-AMPKα was performed by ImageJ and normalized by AMPKα. *P < 0.05.

It has been reported that NTSR3 forms a complex with NTSR1 and modulates NTSR1-mediated signaling in the human colorectal cancer cell line HT29 (ref.^35^). To confirm this interaction, we performed IP in HEK-293 cells stably co-overexpressing GFP-NTSR1/RFP-NTSR3 using GFP antibody. RFP-NTSR3 was detected by RFP antibody in cells co-overexpressing GFP-NTSR1/RFP-NTSR3 but not in cells overexpressing the GFP vector (Fig. 1a, left). NTS treatment appeared to slightly reduce the interaction (Fig. 1a, left). Analysis of the input of the same cell lysates demonstrated co-overexpression of GFP-NTSR1/RFP-NTSR3 using GFP and RFP antibodies (Fig. 1a, right). As shown in Fig. 1b, the majority of GFP-NTSR1 localized on the membrane in the control cells; NTS treatment caused internalization of GFP-NTSR1 into the cytosol at 60 min and was not apparent at 120 min; however, in cells overexpressing both GFP-NTSR1 and RFP-NTSR3, GFP-NTSR1 was only weakly detected at 60 min and reappeared on the membrane at 120 min, suggesting the promoting effect of NTSR3 in NTSR1 trafficking. In GFP-NTSR1 cells (Fig. 1c), phosphorylation of ERK1/2 (p-ERK1/2), an activation marker of NTS–NTSR1 signaling^36^, was dramatically stimulated by NTS treatment at 15 min. With overexpression of both GFP-NTSR1 and RFP-NTSR3, NT-stimulated p-ERK1/2 was noted at 15, 30 and 60 min, which was similar to the effect noted with GFP-NTSR1 overexpression alone; however, p-ERK1/2 was increased at 120 min, further suggesting that NTSR3 facilitates NTSR1 intracellular trafficking. Therefore, we confirm the physical association of NTSR1 and NTSR3 in NTS–NTSR1 signaling.

We found *Ntsr1* mRNA to be expressed in jejunal mucosa of *Ntsr1*^*+/+*^ mice, which was about 20-fold lower compared with *Ntsr1*^*+/+*^ mouse brain *Ntsr1* mRNA levels (Supplementary Fig. 1, red bars), whereas there was no detection of *Ntsr1* mRNA in *Ntsr1*^*−/−*^ mice (Supplementary Fig. 1, blue bars). We further validated these results in jejunal organoids (Fig. 1d). NTS treatment consistently activated p-ERK1/2 in jejunal organoids isolated from *Ntsr1*^*+/+*^ mice (Fig. 1e). Only a minimal basal level of p-ERK1/2 was noted in *Ntsr1*^*−/−*^ organoids, and as expected, NTS treatment failed to induce p-ERK1/2 (Fig. 1e). Notably, the phosphorylated AMPKα (p-AMPKα) was inhibited by NTS treatment in *Ntsr1*^*+/+*^ organoids, although statistical analysis failed to detect significance, and the levels of p-AMPKα in *Ntsr1*^*−/−*^ organoids were significantly higher than *Ntsr1*^*+/+*^ organoids treated with NTS (Fig. 1e). Together, we demonstrate the expression of NTR1 in IECs; NTS inhibits p-AMPKα expression in JECs jejunal epithelial cells.

### Nts deficiency improves AMPK signaling impaired by HFD feeding

**Fig. 2:**
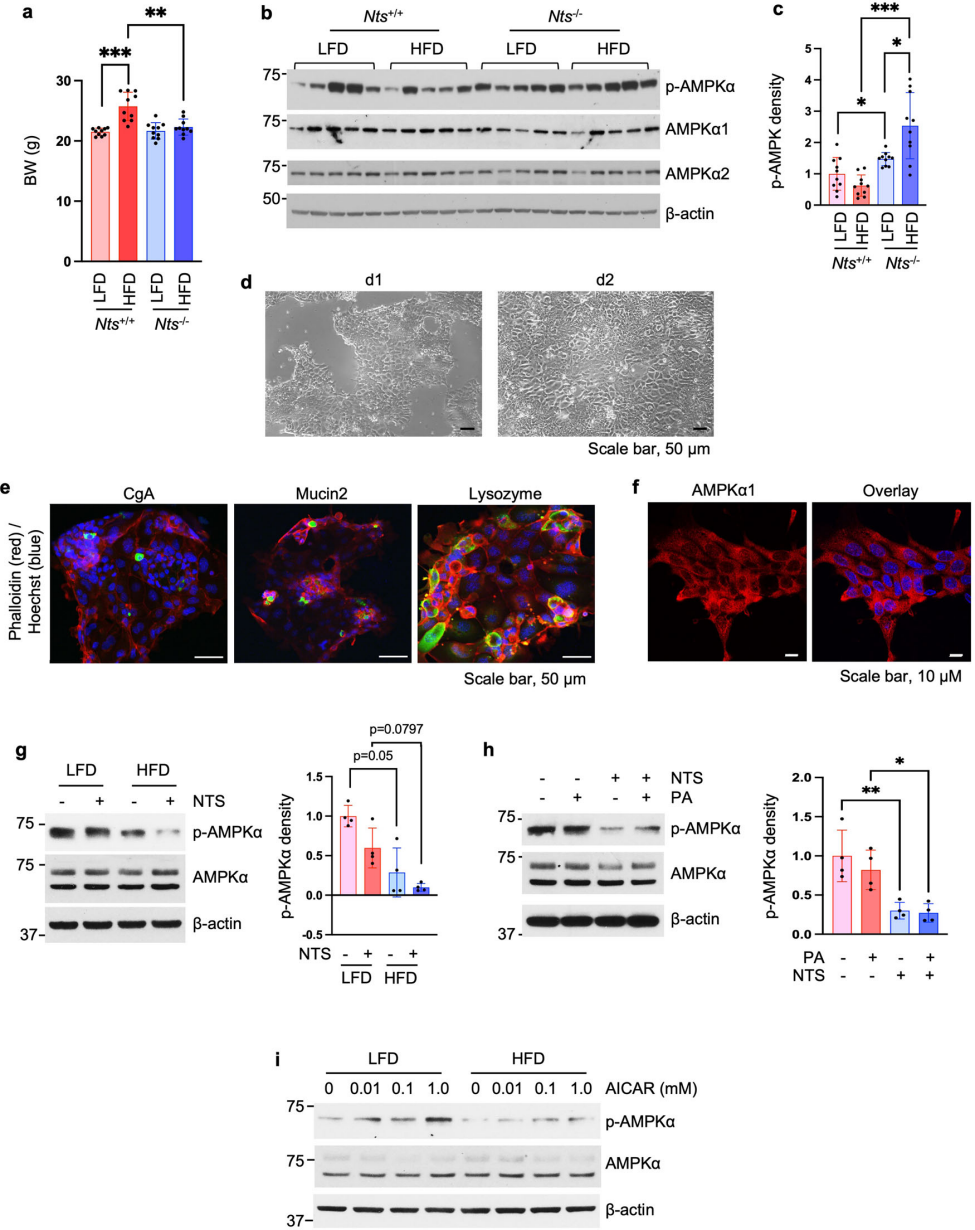
NTS deficiency reverses HFD-inhibited p-AMPK expression. **a**–**c** Male mice were fed either a LFD or a HFD for 6 weeks at weaning. BW was measured before mice were euthanized (**a**). Protein was extracted from jejunal mucosal scrapings and analyzed by western blot, and data show representative results from five mice per group (**b**). Densitometric analysis of p-AMPKα was performed by ImageJ and normalized by the average of AMPKα1/α2 (**c**). *n* = 10 mice per group. *P < 0.05, **P < 0.01, ***P < 0.001. **d** Images of jejunal monolayers were taken by inverted microscope at d1 and d2. **e** IF of jejunal monolayers at d2 and counterstained with Hoechst and phalloidin. **f** IF of jejunal monolayers at d2 using anti-AMPKα1 antibody (red) and counterstained with Hoechst (blue). **g** Western blot of proteins isolated from jejunal monolayers from male WT mice fed a LFD or HFD for 6 weeks at weaning; cells were treated with or without NTS (10 nM) for 10 min. Densitometric analysis of p-AMPKα was performed by ImageJ and normalized by AMPKα. *n* = 4 mice per group. **h** Male WT mice (4 months old) were maintained on NC. Western blot analysis of protein isolated from jejunal monolayers treated with NTS (10 nM) or PA (30 μM) alone or in combination for 24 h. Densitometric analysis of p-AMPKα was performed by ImageJ and normalized by AMPKα. *n* = 4 mice per group. *P < 0.05, **P < 0.01. **i** Male WT mice were fed a LFD or HFD for 6 weeks at weaning. Jejunal crypts were isolated for monolayer culture; cells were treated with AICAR at different concentrations for 24 h. *n* = 3 mice per group.

To extend findings from our previous study^7^, we further assessed the inhibitory effects of NTS on AMPK activity in association with obesity. Male mice were fed a LFD or a HFD for 6 weeks at weaning. HFD feeding significantly increased BW in *Nts*^+/+^ mice compared with the LFD (Fig. 2a). BW gain in HFD-*Nts*^+/+^ mice was prevented in *Nts*^−/−^ mice fed a HFD (Fig. 2a). In *Nts*^+/+^ mice fed a HFD, p-AMPKα was downregulated in jejunal mucosal scrapings compared with *Nts*^+/+^ mice fed a LFD, although this failed to reach statistical significance (*P* = 0.0761) (Fig. 2b, c). Higher basal levels of p-AMPKα were noted in *Nts*^−/−^ mice compared with *Nts*^+/+^ mice fed a LFD (Fig. 2b, c). Importantly, the decrease in p-AMPKα observed in *Nts*^+/+^ mice fed HFD was reversed in *Nts*^−/−^ mice fed a HFD (Fig. 2b, c), suggesting that NTS deficiency improves p-AMPKα expression associated with feeding a HFD.

### NTS and PA inhibit p-AMPKα and HFD reduces AMPK responsiveness

For the ex vivo experiments, we established two-dimensioinal JEC monolayer models based on published protocols^32^ with modifications as described in the ‘Materials and methods’ section. As shown in Fig. 2d, isolated crypts formed monolayers on d1 and expanded on d2. EE, goblet and Paneth cells were detected by IF staining (Fig. 2e). We next detected the expression pattern of AMPKα1 in the JEC monolayer cells and found that the majority of AMPKα1 was localized in the cytosol and less in the nucleus (Fig. 2f). As shown in Fig. 2g, the HFD decreased p-AMPKα expression in JECs compared with LFD-fed mice (*P* = 0.05). NT treatment slightly inhibited p-AMPKα expression in JECs from mice fed LFD; this effect was pronounced in JECs from mice fed a HFD, although statistical analyses failed to detect significance. Again, in JECs from WT mice, less effect of PA alone on p-AMPKα was noted; treatment with NTS alone significantly decreased p-AMPKα; compared with PA treatment alone, PA decreased p-AMPKα expression in the presence of NTS (Fig. 2h). Furthermore, treatment with AICAR increased p-AMPKα in a dose-dependent fashion in JECs isolated from WT mice fed a LFD; however, AICAR-activated p-AMPKα expression was attenuated in JECs isolated from mice fed a HFD (Fig. 2i). Together, these findings further demonstrate a decreased AMPK signaling in obese JECs and show that NTS contributes to the impaired AMPK signaling in this condition.

### NTS deficiency improves AMPK and insulin signaling pathways with aging and HFD feeding

**Fig. 3:**
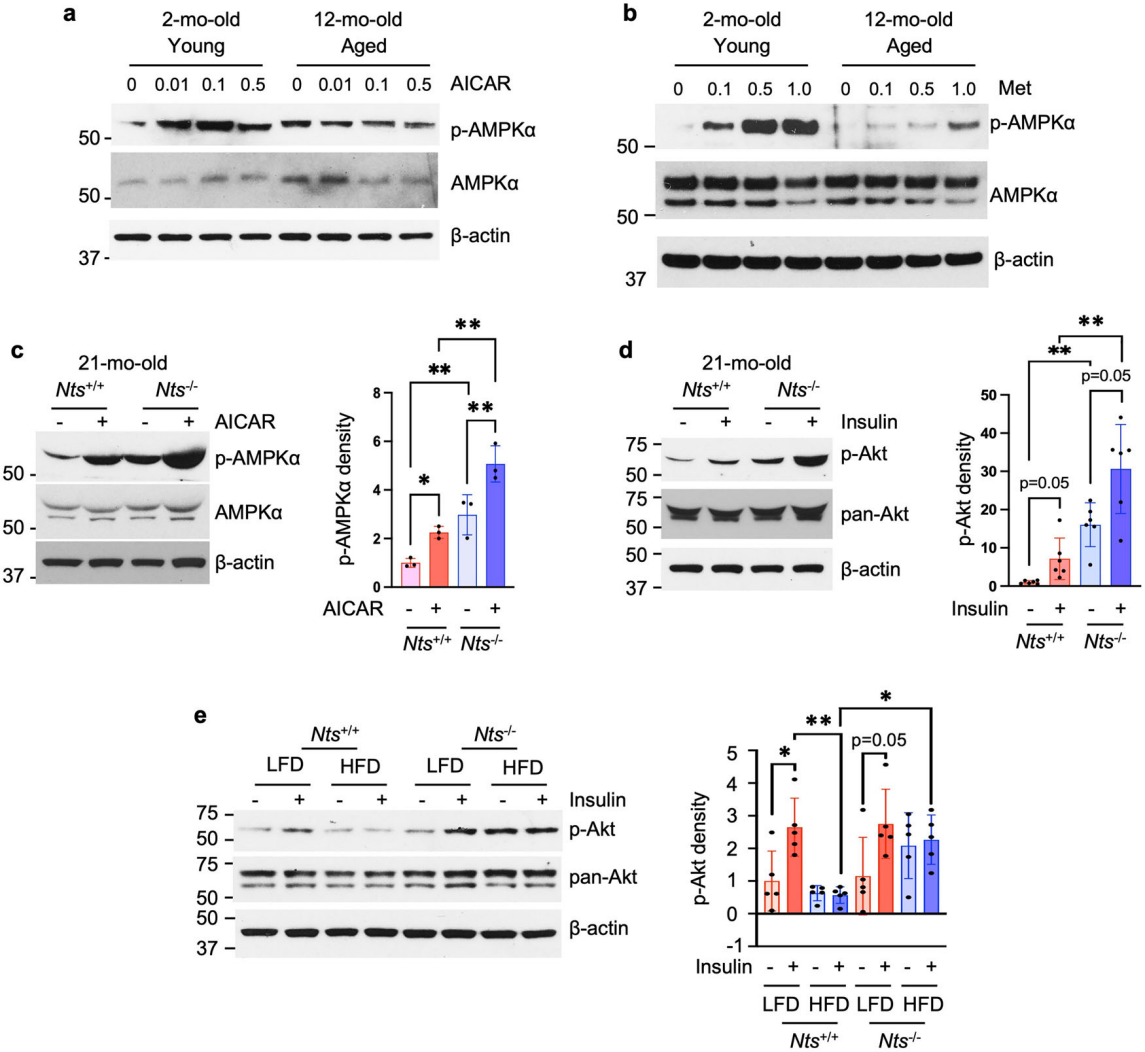
NTS deficiency improves AMPK and insulin signaling associated with aging and HFD feeding. **a**, **b** Western blot analysis of protein extracted from male young and aged jejunal monolayers treated with AICAR (**a**) or Met (**b**) at different concentrations for 24 h. *n* = 3 mice per group. **c**, Jejunal monolayers from male mice on NC were treated with 1 mM AICAR for 24 h followed by western blot. Densitometric analysis of p-AMPKα was performed by ImageJ and normalized by AMPKα. *n* = 3 mice per group. *P < 0.05, **P < 0.01. **d** Jejunal monolayers from male mice on NC were treated with or without insulin (100 nM) for 10 min followed by western blot. Densitometric analysis of p-Akt was performed by ImageJ and normalized by pan-Akt. *n* = 6 mice per group. **P < 0.01. **e** Jejunal crypts were isolated from male mice fed either a LFD or a HFD for 6 weeks at weaning, and monolayers formed; cells were treated with or without (100 nM) for 10 min. Densitometric analysis of p-Akt was performed by ImageJ and normalized by pan-Akt. *n* = 5 mice per group. *P < 0.05, **P < 0.01.

Whether AMPK activity is altered in enterocytes with aging remains unknown. We found that AICAR treatment increased p-AMPKα in young but not aged JECs, demonstrating the abnormal AMPK signaling in response to stimulation in aging JECs (Fig. 3a). Similarly, Met dramatically activated p-AMPKα in young but only slightly in aged JECs, further confirming the defective AMPK signaling in aged enterocytes (Fig. 3b). The higher basal levels of p-AMPKα were noted in aged *Nts*^−/−^ cells compared with *Nts*^+/+^, and, more importantly, treatment with a higher concentration of AICAR (1 mM) increased p-AMPKα in aged *Nts*^+/+^ JECs, which was further enhanced in *Nts*^−/−^ cells (Fig. 3c), demonstrating that AMPKα responsiveness was increased in the absence of NTS.

Decreases in AMPK activity in abdominal subcutaneous, omental and epiploic adipose tissue of individuals with obesity are associated with insulin resistance^37^; activation of AMPK increases insulin sensitivity^38^. In aged enterocytes, basal and insulin-stimulated phosphorylation of Akt (p-Akt) was enhanced in JECs from *Nts*^−/−^ mice compared with JECs from *Nts*^+/+^ mice (Fig. 3d). As shown in Fig. 3e, insulin stimulated p-Akt in JECs from *Nts*^+/+^ mice fed a LFD. However, insulin failed to stimulate p-Akt in JECs from *Nts*^+/+^ mice fed a HFD; the levels of insulin-stimulated p-Akt in JECs from HFD-fed *Nts*^−/−^ mice were higher compared with HFD-fed *Nts*^+/+^ mice. Therefore, both AMPK and insulin signaling were defective in enterocytes isolated from obese and aged WT mice; however, NTS deficiency improved AMPK and insulin signaling, suggesting a role of NTS in the development of obesity, insulin resistance or aging through the downregulation of AMPK and insulin signaling pathways.

### NTS deficiency prevents the increase of FABP1 expression induced by HFD feeding

**Fig. 4:**
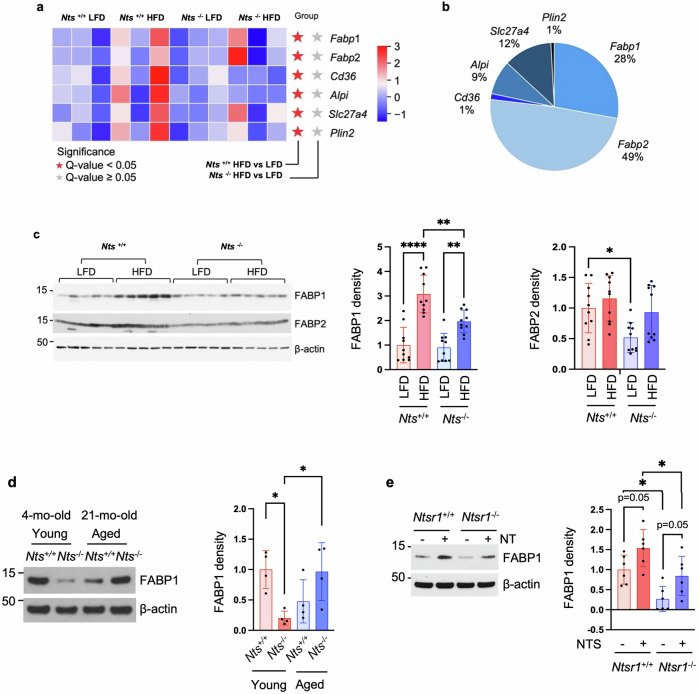
NTS deficiency reverses abnormal FABP1 expression induced by HFD feeding and aging. **a**, **b** Female mice were fed a LFD or HFD for 28 weeks at weaning. *n* = 3 mice per group. The heat map of RNA-seq data shows the individual gene expression among four groups in jejunal mucosal scrapings (**a**). The percentage portion is plotted from RNA-seq data of WT mice fed a LFD (**b**). **c** Western blot analysis of protein extracted from jejunal mucosal scrapings of mice as in Fig. 2a; data show representative results from five mice per group. Densitometric analysis of FABP1 and FABP2 was performed by ImageJ and normalized by β-actin. *n* = 10 mice per group. *P < 0.05, **P < 0.01, ****P < 0.0001. **d** Western blot analysis of protein extracted from jejunal monolayers of male young and aged *Nts*^+/+^ and *Nts*^−/−^ mice fed NC. Densitometric analysis of FABP1 was performed by ImageJ and normalized by β-actin. *n* = 4 mice per group. *P < 0.05. **e** Western blot analysis of protein extracted from jejunal monolayers of male *Ntsr1*^+/+^ and *Ntsr1*^−/−^ mice fed NC at 4 months of age. Densitometric analysis of FABP1 was performed by ImageJ and normalized by β-actin. *n* = 6 mice per group. *P < 0.05.

Genes involved in small intestinal lipid metabolism play important roles in the physiological regulation of whole-body energy homeostasis and are changed by adaptation in response to HFD feeding^39^. From the RNA-seq analysis of the jejunal mucosal scrapings, we found that genes involved in intestinal lipid metabolism (*Fabp1*, *Fabp2*, *Cd36*, *Alpi*, *Fatp4* and *Plin2*) were significantly increased by HFD feeding in *Nts*^+/+^ mice compared with *Nts*^+/+^ mice fed a LFD, which was not noted in *Nts*^−/−^ mice fed a HFD (Fig. 4a). From the expression abundance of those genes in the RNA-seq data, *Fabp2* (49%) and *Fabp1* (28%) are the major genes (Fig. 4b).

We further assessed the protein expression of FABP1 and FABP2 in association with HFD feeding as well as aging. As shown in Fig. 4c (left and middle), western blot analysis of jejunal mucosal scrapings showed that FABP1 protein expression was elevated by HFD feeding in *Nts*^+/+^ mice (3.1-fold); this effect was reduced in *Nts*^−/−^ mice (1.7-fold). A significant decrease of FABP2 was noted in *Nts*^−/−^ mice versus *Nts*^*+/+*^ mice (Fig. 4c, left and right). From our data, it appears that FABP1 is the critical player in the development of obesity and is affected by NTS. Therefore, we focused on FABP1 in the following experiments.

We also evaluated whether NTS affected FABP1 levels in aging. As shown in Fig. 4d, FABP1 protein expression was inhibited in the JEC monolayer cells from young *Nts*-deficient mice. The expression of FABP1 was relatively lower in cells from aged *Nts*^+/+^ mice compared with cells from young *Nts*^+/+^ mice; this reduction was rescued by NTS deficiency. We further confirmed that NTS increased FABP1 expression in *Ntsr1*^*+/+*^ JEC monolayers, which was attenuated in the jejunal monolayers collected from *Ntsr1*^−/−^ mice (Fig. 4e). Thus, we provide evidence showing the upregulation of FABP1 by HFD feeding, which was attenuated by NTS deficiency. FABP1 expression is reduced in young *Nts*^−/−^ JECs. Aging causes a reduction of FABP1 in enterocytes, whereas aging-decreased FABP1 expression can be prevented in *Nts*^−/−^ JECs. FABP1 is probably increased by compensatory mechanisms in response to HFD consumption, and this compensation may contribute to the development of obesity; thus, we postulate that NTS deficiency inhibits the compensatory effects and, in so doing, attenuates the associated condition of obesity.

### AMPK activity is negatively correlated with FABP1 expression

**Fig. 5:**
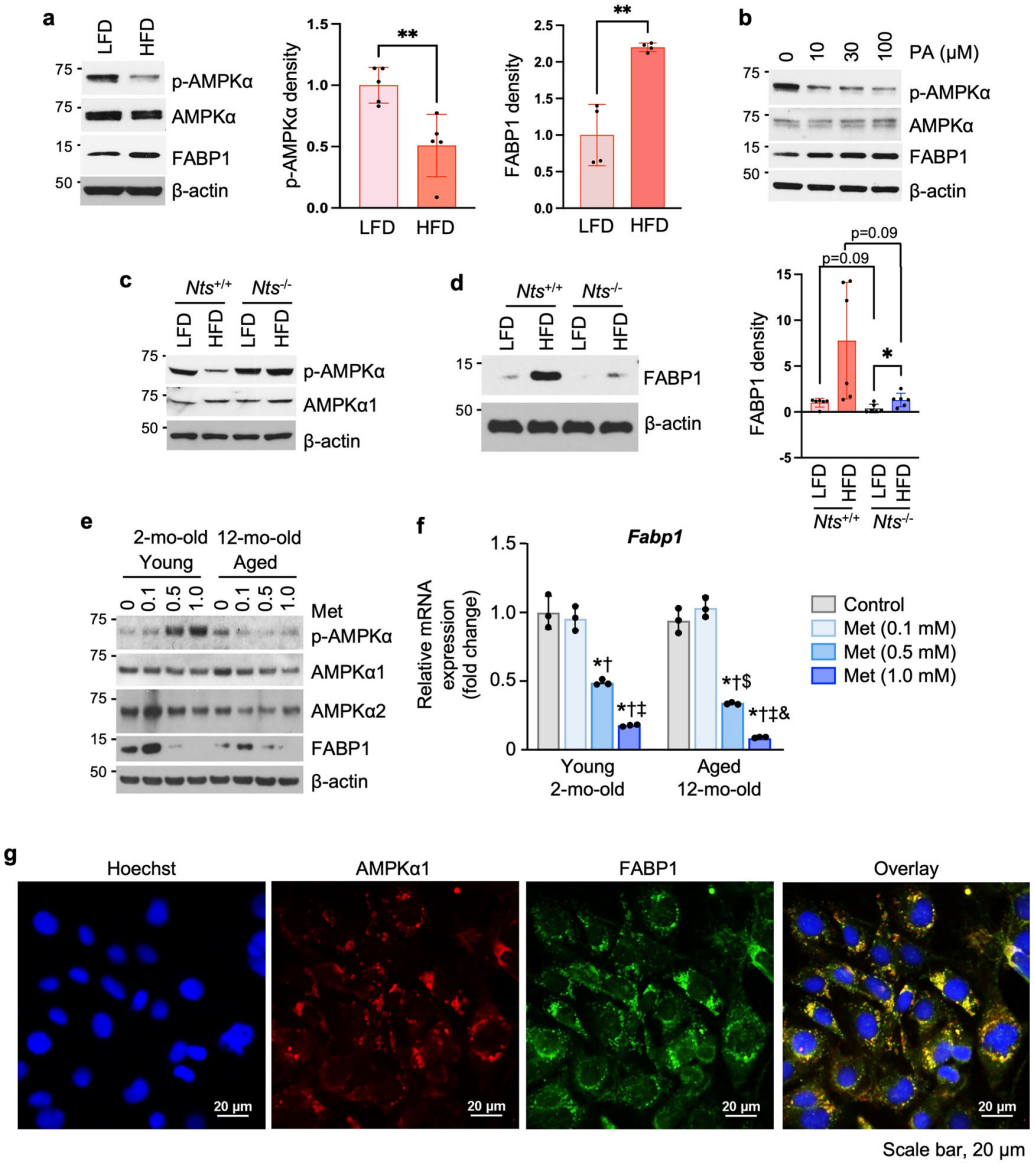
AMPK activity is correlated with FABP1 expression. **a** Western blot analysis of protein extracted from monolayers of male WT mice fed a LFD or HFD for 6 weeks at weaning. Densitometric analysis of p-AMPKα (*n* = 5 mice per group) and FABP1 (*n* = 4 mice per group) was performed by ImageJ and normalized by AMPKα and β-actin, respectively. **P < 0.01. **b** Western blot analysis of protein extracted from monolayers of male WT mice (4 months old); cells were treated with PA at different concentrations for 24 h. **c**, **d** Western blot analysis of protein extracted from jejunal monolayers of male *Nts*^+/+^ and *Nts*^−/−^ mice fed a LFD or HFD for 6 weeks at weaning. Densitometric analysis of FABP1 was performed by ImageJ and normalized by β-actin. (*n* = 6 mice per group). *P < 0.05. **e**, **f** Western blot (**e**) and qPCR (**f**) analysis of protein and RNA, respectively, extracted from monolayers of male young and aged mice on NC; cells were treated with Met at different concentrations for 24 h. *n* = 3 mice per group. *P < 0.001 versus control in young and aged, respectively; ^†^*P* < 0.001 versus 0.1 mM in young and aged, respectively; ^‡^*P* < 0.001 versus 0.5 mM in young and aged, respectively; ^$^*P* < 0.01 versus young 0.5 mM; ^&^*P* < 0.001 versus young 1.0 mM. **g** IF staining of IEC-6 cells; images were taken by confocal microscopy.

We next determined the regulation of AMPK on FABP1 expression. p-AMPKα levels were inhibited by the HFD (Fig. 5a, left and middle), whereas FABP1 expression was increased in JECs from WT mice fed a HFD (Fig. 5a, left and right). Consistently, PA treatment decreased p-AMPKα but increased FABP1 protein expression in a dose-dependent fashion (Fig. 5b). Again, decreased p-AMPKα was noted in JECs from *Nts*^+/+^ mice fed a HFD, but this reduction was prevented in JECs from *Nts*^−/−^ mice fed the same diet (Fig. 5c). The decrease in p-AMPKα in the JECs from *Nt*^+/+^ mice fed a HFD was accompanied by a noticeable increase in FABP1 protein expression, although statistical analysis failed to detect significance (Fig. 5d). HFD-increased FABP1 expression was reversed in JECs from *Nts*-deficient mice fed a HFD (*P* = 0.09) (Fig. 5d).

### AMPK activation decreases FABP1 expression

We next used AMPK activators to stimulate AMPK activity and then evaluated their effects on FABP1 expression in young and aged JECs. As shown in Fig. 5e, Met treatment activated p-AMPKα in JECs from young mice but not from the aged mice; basal FABP1 expression was decreased in cells of aged mice compared with cells collected from young mice; Met inhibited FABP1 expression in both young and aged JECs. Furthermore, Met treatment decreased mRNA expression of *Fabp1* in the JECs from both young and aged mice (Fig. 5f). Consistent with the effect noted with Met, AICAR treatment dramatically decreased FABP1 protein expression in a dose-dependent fashion in JECs from young mice (4 months old) (Supplementary Fig. 2a).

We also evaluated the effects of AICAR on FABP1 expression in JECs isolated from *Nts*^+/+^ and *Nts*^−/−^ mice fed a HFD. AICAR treatment had only a minimal effect on FABP1 expression in JECs from HFD-fed *Nts*^+/+^ mice; however, a decrease of FABP1 expression was noted in HFD-fed *Nts*^−/−^ JECs (Supplementary Fig. 2b), indicating a correlation of FABP1 regulation with AMPK activity that is blunted by HFD feeding but improved in the absence of NTS. Interestingly, we found the cytosolic colocalization of AMPKα1 with FABP1 in IEC-6 cells (Fig. 5g), a rat normal small intestinal epithelial cell line, which has been used in FA uptake studies^40^. These findings demonstrate that Met and AICAR decrease FABP1 expression.

### NTS inhibits p-AMPKα and increases FABP1 expression in human IECs

**Fig. 6:**
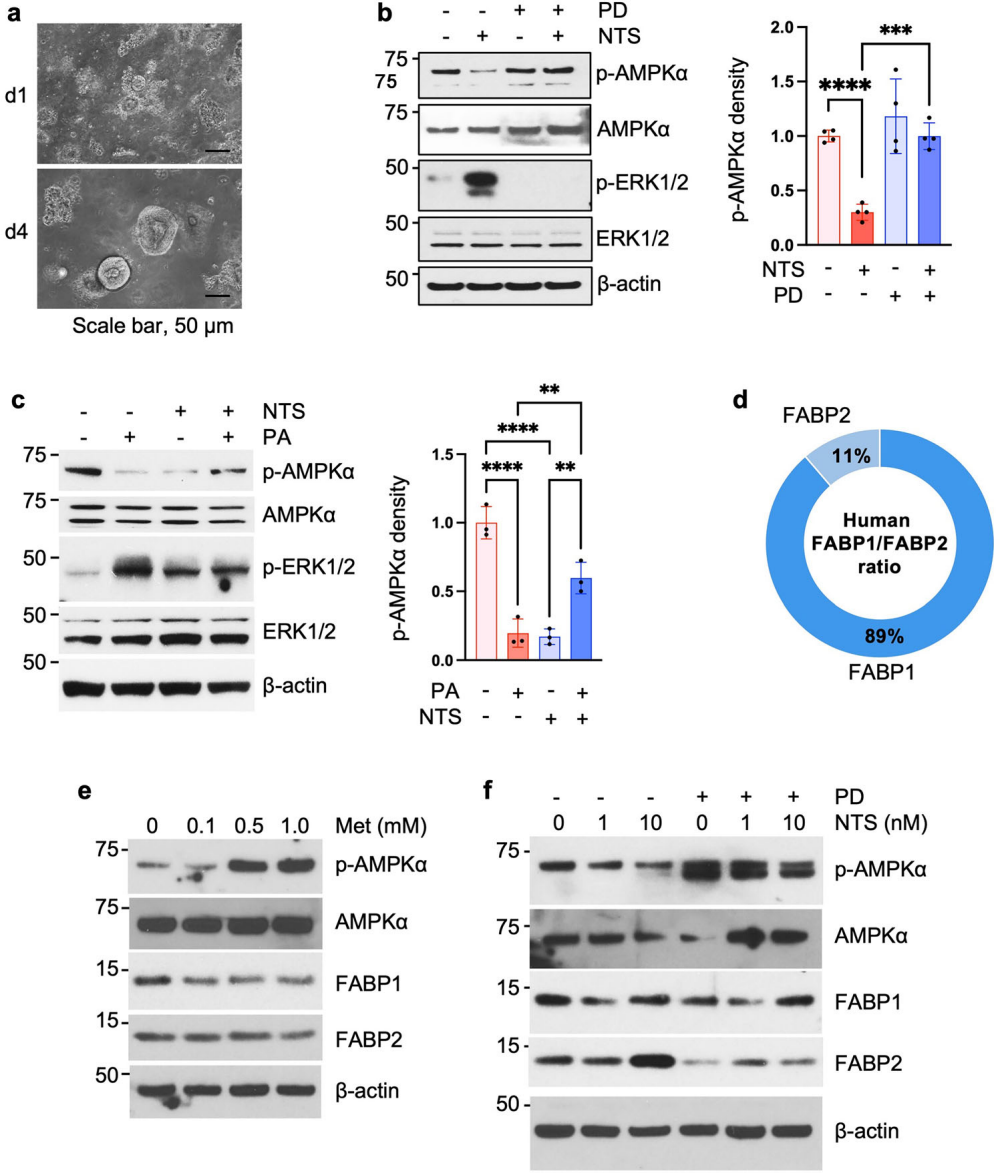
Regulation of NT on AMPK expression in human IEC organoids. Human duodenal crypts were isolated, and organoids were established. **a** Images were taken at d1 and d4 by an inverted microscope. **b** Western blot analysis of protein extracted from duodenal organoids pretreated with PD (2.5 μM) for 30 min followed by treatment with or without NTS (10 nM) for 10 min. Densitometric analysis of p-AMPKα was performed by ImageJ and normalized by AMPKα. *n* = 4 cases per group. ***P < 0.001, ****P < 0.0001. **c** Western blot analysis of protein extracted from duodenal organoids treated at seeding with or without NTS (10 nM) or PA (10 μM) alone or combined for 7 days. Densitometric analysis of p-AMPKα was performed by ImageJ and normalized by AMPKα. *n* = 3 cases per group. **P < 0.01, ****P < 0.0001. **d** The percentage of the portion of FABP1 versus FABP2 by qPCR analysis of RNA extracted from duodenal organoids. **e**, **f** Western blot analysis of protein extracted from duodenal organoids treated with Met at different concentrations (**e**) or NTS (0, 1 and 10 nM) in the presence or absence of PD (2.5 μM) (**f**) for 24 h.

To further confirm our findings in mice, we next determined the effects of NTS on human intestinal epithelial cells established as organoid cultures from normal duodenal samples after surgical resection (Fig. 6a). Organoids were treated with or without NTS (10 nM) for 10 min in the presence or absence of an MEK inhibitor, PD 98059 (2.5 μM). As shown in Fig. 6b, NTS treatment activated p-ERK1/2 and inhibited p-AMPKα; PD 98059 completely inhibited p-ERK1/2 and reversed NTS-inhibited p-AMPKα expression. PA treatment suppressed p-AMPKα (Fig. 6c), which mimicked the effects of the HFD on p-AMPKα in mice. NTS treatment decreased p-AMPKα, which was increased by the combination of PA and NTS (Fig. 6c).

In contrast to mice, FABP1 is the predominant isoform in human duodenal organoids compared with FABP2 as evaluated by qPCR (Fig. 6d). Met treatment of human duodenal organoids increased p-AMPKα expression and decreased FABP1 (Fig. 6e). Minimal inhibition of FABP2 protein expression by Met was detected only at 1 mM (Fig. 6e). NTS decreased p-AMPKα at 1 and 10 nM; this reduction was prevented in the presence of the PD compound (Fig. 6f). NTS treatment attenuated FABP1, which was not significantly affected by PD; however, NTS increased FABP2 expression at 10 nM, and both basal and NT-enhanced FABP2 expression was blocked by PD 98059 (Fig. 6f). Thus, similar to the negative regulation of AMPK activation by NTS in mice, we show that NTS inhibits AMPK activity in human IECs. However, Met significantly inhibits FABP1, but not FABP2 protein expression, which differs from mice in that either Met or AICAR inhibits both FABP1 and FABP2. These results suggest that FABP2 expression is more sensitive to NTS treatment compared with FABP1 in human IECs.

### NTS reduces lifespan

**Fig. 7:**
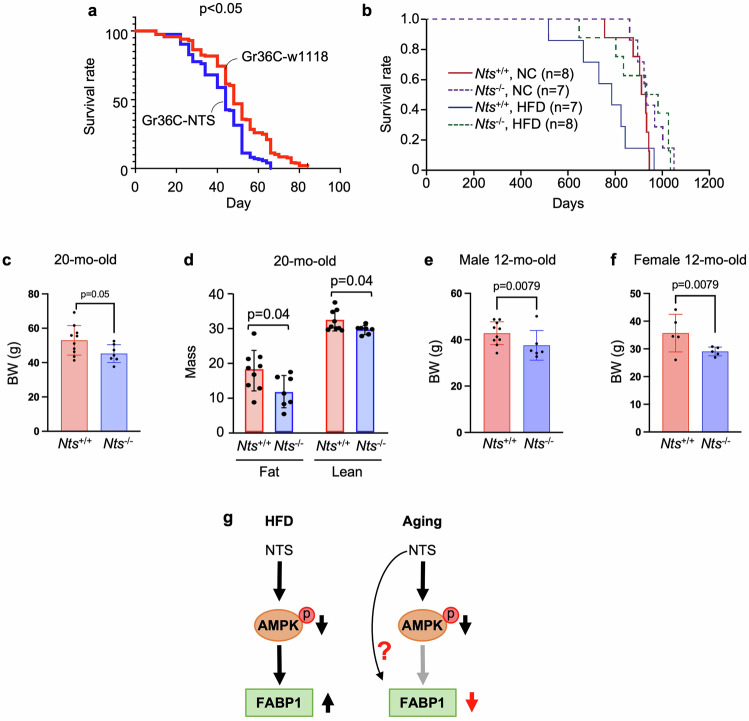
NTS negatively alters lifespan in *Drosophila* and mice. **a** The lifespan of adult male flies with different genetic backgrounds. The eighth generation of flies synchronized with the *w1118* strain was used. Gr36C-w1118, *n* = 344 flies; Gr36C-NTS, *n* = 238 flies. **b** The lifespan of male *Nts*^+/+^ and *Nts*^−/−^ mice maintained on NC or HFD at weaning until they died. *Nts*^+/+^ NC, *n* = 8; *Nts*^+/+^ HFD, *n* = 7, *Nts*^−/−^ NC; *n* = 7, *Nts*^−/−^ HFD, *n* = 8. *Nts*^+/+^ HFD versus NC, *P* = 0.0276, *Nts*^−/−^ HFD versus NC, *P* = 0.8185, *Nts*^−/−^ versus *Nts*^+/+^ on NC or HFD, *P* = 0.0106 (Wilcoxon test). **c**, **d** The BW of male mice at 20 months of age maintained on NC (**c**). Fat and lean mass (**d**) was measured by EchoMRI on the mice in **c**. *Nts*^+/+^, *n* = 10 mice per group; *Nts*^−/−^, *n* = 7 mice per group. **e**, **f** The BW of 12-month-old male (**e**) and female (**f**) mice. Male *Nts*^+/+^, *n* = 10 mice per group; male *Nts*^−/−^, *n* = 7 mice per group. Female *Nts*^+/+^, *n* = 5 mice per group; female *Nts*^−/−^, *n* = 5 mice per group. **g** A summary of the current study.

The intestine plays a pivotal role in lifespan regulation and coordinating organismal aging by linking nutrient metabolism, immune function and interorgan communication^41^. Previously, we reported an increase in the level of both ileal mucosal NTS mRNA and plasma NTS with aging in rats, indicating the association of NTS with aging^42^. Therefore, we determined whether NTS contributes to reduced lifespan through inhibition of AMPK activity in the gut. We first utilized the *Drosophila* model, in which human full-length NTS cDNA was expressed in midgut EE cells as described in our previous study^7^. As shown in Fig. 7a, NTS overexpression reduced male *Drosophila* lifespan compared with control flies expressing *Gr36C-w1118* (*P* < 0.05). Female flies showed similar results (*P* < 0.0001) (Supplementary Fig. 3); however, it appears that NTS has a more pronounced effect on the lifespan of female flies versus male flies. Other studies suggest that NTS has different effects between males and females, with females appearing to be more sensitive to the effects of NTS, particularly in relation to stress, anxiety and eating behaviors, probably due to the influence of sex hormones on NTS expression and function^43–45^.

Studies have shown that obesity is associated with increased mortality at all ages, for both men and women^46^. We found that the lifespan was shortened in male *Nts*^+/+^ mice fed a HFD compared with NC-fed mice (Fig. 7b). Importantly, NTS deficiency extended the lifespan compared with WT littermates under both normal conditions and conditions of obesity (Fig. 7b). NTS deficiency decreased weight gain and fat mass in aged mice (Fig. 7c, d). Decreased BW by NTS deficiency was also noted in 12-month-old male and female mice (Fig. 7e, f). Thus, our results suggest that NTS negatively contributes to lifespan. However, the precise role of NTS in lifespan reduction remains complex. The hypothalamic–pituitary–adrenal axis is a key neuroendocrine system that regulates the body’s response to stress, metabolism, immune function and circadian rhythms^47^. Elevated NTS levels have been linked to hyperactive hypothalamic–pituitary–adrenal axis responses leading to excessive cortisol secretion, which may contribute to chronic stress, neuroinflammation and mood disorders^45^. It is possible that NTS affects the HPA hypothalamic-pituitary-adrenal (HPA) axis, which might contribute to lifespan. From our observations, NTS affects intestinal AMPK and insulin signaling and lipid absorption, which contribute to overall health and lifespan.

## Discussion

Accumulated evidence has demonstrated the importance of AMPK in maintaining small intestinal homeostasis^48^. However, whether AMPK plays a role in regulation of intestinal lipid absorption remain controversial, especially under the conditions of obesity and aging. In the current study utilizing a *Drosophila* model overexpressing NTS in gut EE cells, NTS- and NTSR1-KO mouse models as well as ex vivo models, such as mouse jejunal epithelial monolayers and organoids and human duodenal organoids, and the rat normal small intestinal epithelial cell line IEC-6, we found that (1) AMPK and insulin signaling was inhibited by HFD feeding and aging in WT mice but not in NTS-deficient mice; (2) FABP1 expression was increased by a HFD but decreased with aging, which was rescued in NTS-deficient mice; (3) AICAR or Met activated AMPK and concurrently decreased FABP1; (4) AMPKα1 colocalized with FABP1 in the cytosol of IEC-6 cells; and (5) overexpressing NTS in gut EE cells reduced *Drosophila* lifespan, whereas NTS deficiency extended the lifespan of mice.

NTS–NTSR1 signaling can activate ERK1/2 signaling^36^. We previously found that NTS enhanced, while AICAR inhibited, FA uptake and that AICAR-inhibited FA uptake was abrogated in the presence of NTS^7^. ERK1/2 inhibition prevents FA uptake in rat cardiac myocytes, indicating that ERK1/2 signaling plays a positive role in FA uptake^49^. Ca^2+^ mediates the activation of NTS–NTSR1–ERK1/2 signaling; thus, these findings are consistent with our previous^7^ and current findings that NTS–NTSR1–ERK1/2 signaling promotes FA uptake in small intestinal epithelial cells. Excessive lipids associated with obesity induce mitochondrial overactivation in an effort to prevent lipid accumulation and lipotoxicity by enhancing FA oxidation, as shown in muscle, liver and brown fat^50^. The overactivation of mitochondria leads to a large amount of ATP, which inactivates AMPK to suppress glucose uptake and inhibit ATP production^50^. In the case of Met as well as other insulin-sensitizing agents, such as TZDs Thiazolidinediones and berberine, mitochondrial overactivation was inhibited, resulting in decreased ATP production and AMPK activation^51–55^. Therefore, we speculate that this may be the underlying mechanism for the inhibition of AMPK by NTS and HFD feeding.

We found that HFD feeding increased FABP1 but not FABP2 protein expression in the jejunal mucosa; this increased expression was prevented by NTS deficiency, suggesting a positive contribution of NTS in HFD-upregulated FAPB1 expression. However, we detected an increase of FABP1 only in NTS-treated jejunal organoids derived from *Ntsr1*^+/+^ mice; this effect was attenuated in *Ntsr1*^−/−^ mice. These data indicate that NTS–NTSR1 signaling is more likely to regulate small intestinal lipid metabolism through FABP1 and not through FABP2. Both FABP1 and FABP2 are found to be largely expressed in the jejunal absorptive villus cells, but not in crypt cells^56^, demonstrating their function in small bowel lipid absorption. The potential and specific functions of FABP1 and FABP2 in the enterocyte remain unclear. Although FABP1 and FABP2 are both cytosolic FA-binding proteins and are involved in intracellular trafficking of the FAs, they mediate FA trafficking by different mechanisms. In differentiated Caco-2 cells with FABP1 knockdown cultured on Transwell inserts, apical uptake of oleate and incorporation were decreased^57^; basolateral oleate secretion was also reduced. Garcia, et al.^58^ showed that liver FABP1 was upregulated in an AMPK-activated mouse model. FABP1 possesses an AMPK phosphorylation site and directly interacts with AMPK^58^. In our previous study^7^, we demonstrated the inhibition of AMPK activation by NTS in intestinal epithelial cells. AMPK activity is inversely related to intestinal FA uptake, and NTS promotes FA uptake through the inhibition of AMPK activity. Intraduodenal infusion of Met activated duodenal mucosal AMPK and lowered hepatic glucose production in a HFD-induced rat model of insulin resistance^18^. Intestinal epithelium-specific AMPKα1 KO resulted in weight gain and impaired glucose tolerance compared with WT mice after 6 weeks of HFD feeding^19^. Met fails to ameliorate these metabolic disorders in intestinal AMPKα1-KO mice^19^. Intestinal epithelium-specific AMPKα1 deletion impaired intestinal absorption of long-chain FAs and protected mice from HFD-induced obesity^20^.

In summary, most studies of lipid metabolism in IECs have utilized immortalized cell lines such as Caco-2 cells^59^ as enterocyte models. In our current study (Fig. 7g), we utilized novel IEC monolayers and organoid ex vivo models derived from mice, including *Nts*- and *Ntsr1*-deficient mice, as well as human tissue to demonstrate that NTS contributes to impaired AMPK signaling in IECs associated with HFD-induced obesity. Upregulation of FABP1 is probably an underlying mechanism in mediating HFD-enhanced lipid absorption. Activation of AMPK activity negatively correlates with FABP1 and/or FABP2 expression, providing further evidence that AMPK activity is important in maintaining small intestinal lipid homeostasis. During aging, AMPK activity is decreased. However, FABP1 expression is also decreased, suggesting that different mechanisms are probably involved in NTS-mediated FABP1 expression. Our findings further emphasize the control of lipid absorption in the small intestine by AMPK activation, which might contribute to the prevention of obesity. Moreover, NTS deficiency improves AMPK signaling and FABP1 expression in conditions of obesity and aging.

## Supplementary information

Supplementary Information
