# Supplementary material for: Neurotensin inhibits AMPK activity and concurrently enhances FABP1 expression in small intestinal epithelial cells associated with obesity and aging
Source: Exp Mol Med. 2025 Jun 2;57(6):1189–201. doi: 10.1038/s12276-025-01461-w (PMC12229603; doi:10.1038/s12276-025-01461-w)
Supplement: Supplementary file 1 — Supplementary Information [file 12276_2025_1461_MOESM1_ESM.pdf]

Supplementary Figures

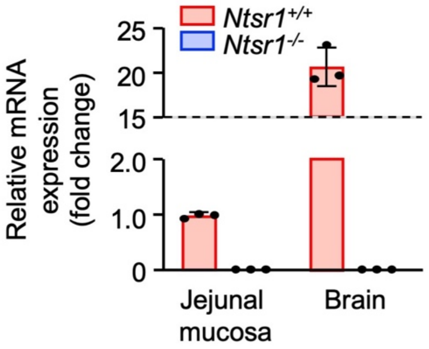

Supplementary Fig. 1. qPCR analysis of jejunal mucosal scrapings from male *Ntsr1*$^{+/+}$ and *Ntsr1*$^{-/-}$ mice (4-mo-old) fed NC. n=3 mice/group. Mouse brain was used as a positive control.

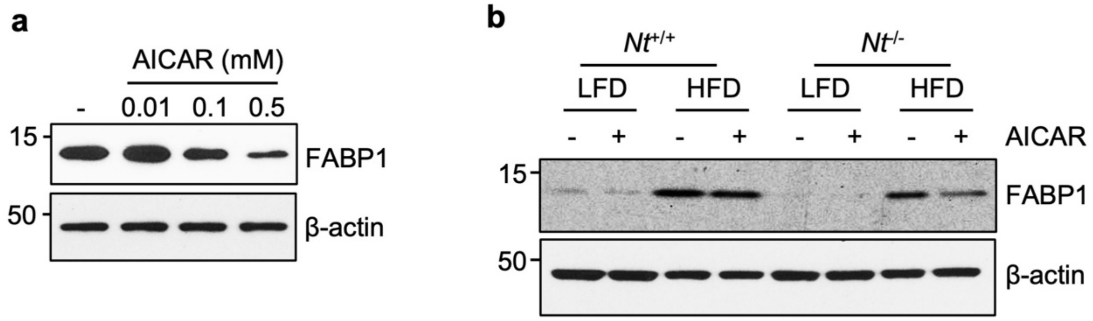

Supplementary Fig. 2. (a) Western blot analysis of protein extracted from jejunal monolayers of male WT mice (4-mo-old) fed NC; cells were treated with AICAR at different concentrations for 24 h. n=3 mice/group. **(b)** Western blot analysis of protein extracted from jejunal monolayers of male mice fed LFD or HFD for 6 wks at weaning; cells were treated with or without AICAR (1 mM) for 24 h.

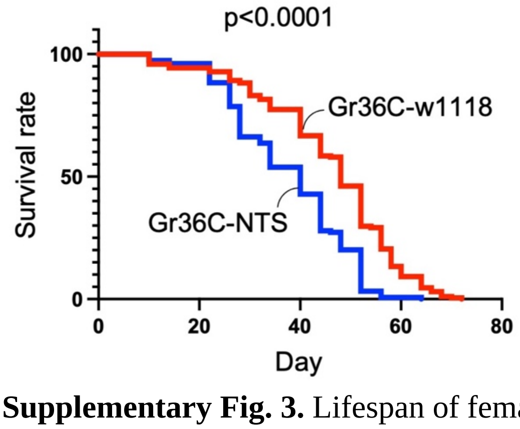

Supplementary Fig. 3. Lifespan of female flies. Gr36C-w1118, n=259; Gr36C-NTS, n=229 flies.
